# The effect of manual therapy on diaphragm function in adults with asthma: Protocol for a randomized controlled trial

**DOI:** 10.12688/f1000research.141455.2

**Published:** 2024-03-19

**Authors:** Dimitrios Tsimouris, Eirini Grammatopoulou, Maria Papandreou, George Gioftsos, George Koumantakis

**Affiliations:** 1 Physiotherapy Department, ,, Egaleo, School of Health & Care Sciences, University of West Attica, 12243, Greece

**Keywords:** Key Words: Asthma; Manual Therapy; Diaphragm; Breathing retraining exercises; Diaphragm mobility, Ultrasound

## Abstract

**Background:**

Diaphragm dysfunction is prevalent among individuals with asthma due to lung hyperinflation and hyperventilation in asthma paroxysm. This study was designed to evaluate the effect of the manual diaphragm release technique (MDRT) on diaphragm function in individuals with asthma.

**Methods:**

Adults with diagnosed stable asthma (n = 24), will be recruited from the General Hospital of Kifissia “Agioi Anargyroi” in Athens, Greece. The volunteers who meet the inclusion criteria will be randomly allocated to two groups: (a) the experimental group (n = 12) that will receive 12 sessions of MDRT in conjunction with breathing retraining exercises (BRE), and (b) the control group (n = 12) that will receive 12 sessions of BRE. Measurements will occur at three time points: before the initiation of treatment sessions (week 0), followed by 12 treatment sessions (week 6), and three months from the beginning of the trial (week 12). The main outcomes will be the diaphragm excursion (ultrasonography) and chest expansion (inch tape), with secondary outcomes the maximal respiratory pressures (digital pressure manometer), dysfunctional breathing (Nijmegen questionnaire), asthma control (ACT), dyspnea (Borg scale) and quality of life (SF-12v2).

**Discussion:**

The proposed protocol is the first to examine the effectiveness of MRDT on diaphragm’s function in individuals with asthma. Manual Therapy (MT) is a low-cost alternative and supplementary therapy to standard treatment procedures that might improve the biomechanics of respiration in pulmonary rehabilitation.

**Trial Registration:**

Registered on Clinical Trials.gov (ID: NCT05709054)

**Protocol version:**

29/09/2023

**Abbreviations**
ACT: Asthma Control Test
BRE: Breathing Retraining Exercises
COPD: Chronic Obstructive Pulmonary Disease
CWE: Chest Wall Expansion
GOHK: General Oncology Hospital of Kifissia
MDRT: Manual Diaphragm Release Technique
MT: Manual Therapy
NQ: Nijmegen Questionnaire
PR: Pulmonary Rehabilitation
RCT: Randomized Controlled Clinical Trial
US: Ultrasonography
ZOA: Zone Of Apposition

## Introduction

Due to its anatomical structure and contribution to minute ventilation (60–80%), the diaphragm is the most important respiratory muscle. ^1^^–^^3^ An impaired diaphragm is associated with respiratory symptoms such as dyspnea, intolerance to exercise and sleep problems. ^4^

Chronic obstructive pulmonary disease (COPD) and asthma are umbrella terms for various conditions characterized by chronic airway disease. ^5^ COPD and asthma patients frequently experience diaphragmatic dysfunction (DD). ^6^ The diaphragm’s ability to raise and expand the lower ribcage within the zone of apposition (ZOA), where the lower ribcage directly interacts with the diaphragm becomes compromised due to mechanical challenges. This is due to the diaphragm functioning at a disadvantageous shortened position caused by air trapping, which hinders its contraction capacity and increases the respiratory workload. ^7^

In COPD, air progressively remains trapped in the lungs due to airway constriction. The architecture of the thoracic cage is disrupted by this clinical condition during exercise and rest, reducing the diaphragm’s physiological advantage. ^8^^,^^9^ Similarly, Individuals with moderate to severe asthma may have pulmonary overstretching (asthma paroxysm), which can cause functional problems because it reduces expiratory flow (early airway closure), activates inspiratory muscles at the end of expiration, and reduces lung flexibility. ^10^^–^^12^ Although the underlying mechanisms for these two lung conditions, COPD and asthma, differ, both cause secondary complications (pulmonary hyperinflation, hyperventilation syndrome). These features lead to similar pathological changes that impair the diaphragm’s ability to elevate and expand the lower ribcage. ^13^ Consequently, during inspiration, the lower ribcage’s transverse diameter may decrease. ^14^ It’s also important to note that the mechanical disadvantage of the diaphragm in asthma can result in an increased workload for all inspiratory muscles, particularly during exercise, where dynamic hyperinflation may occur, leading to heightened dyspnea. ^15^^,^^16^

Over the past few decades, two research questions have emerged concerning how physiotherapy can enhance the mechanical efficiency of the thorax and the effectiveness of respiratory muscles during breathing in people with obstructive lung diseases. Researchers, from 1990 ^17^ up to 2015, ^18^ have made several hypotheses and implemented physiotherapy interventions to find which procedure is more appropriate to improve the effectiveness of mechanical functioning of the thoracic cage in people with pulmonary diseases. Breathing retraining exercises (BRE) are a widely used, productive method, ^19^^–^^21^ simple, safe, accessible, with a high level of evidence-based efficacy. ^22^^–^^24^

Finally, although specific diaphragm MT techniques have not been documented yet, recent studies have reported evidence for their positive effect on pulmonary rehabilitation (PR). ^25^ In particular, the manual diaphragm release technique (MDRT) aims to directly stretch the muscle fibers of the diaphragm, as detailed in Rochas’ research. ^18^ The study showed an improvement in diaphragm’s mobility, maximum inspiratory pressure (MIP), and exercise capacity (EC) in people suffering from COPD. ^18^ We deem it pertinent to mention that previous studies have demonstrated that even a single MT session can have a positive effect on chest wall mechanics, dyspnea, and peripheral oxygen saturation (SpO2) in individuals with COPD. ^26^^,^^27^ Therefore, dyspnea, being one of the primary symptoms of asthma, can adversely affect both exercise capacity (EC) levels and overall quality of life (QoL). ^28^^–^^31^ According to a study ^26^ a single MT session of soft tissue and joint mobilization immediately improved dyspnea (Borg Scale 0-10, pre: 2.3 ± 0.8 vs 1.8 ± 0.5). The authors reported that the mechanism underlying this improvement could be the increase in respiratory muscle length and thoracic cage flexibility induced by MT, consequently reducing breathing effort and the development of dyspnea in individuals with COPD. ^32^ As for asthma, there is currently no data regarding the efficacy of diaphragm MT methods, except for the pilot study conducted by Macias and colleagues, ^24^ and the study by Elnaggar and colleagues, ^33^ which investigated the efficacy of MDRT in children.

Considering the growing clinical interest in asthma and the recent publications in the field, we believe that a randomized controlled trial (RCT) targeting intervention on the zone of apposition of the diaphragm using the MDRT in adults with asthma for outcomes assessment is warranted.

### Objective of the proposed trial

The primary objective of this study is to explore the impact of MDRT on the diaphragm’s function, particularly on the length-tension relationship and chest wall expansion (CWE) in people suffering from asthma. Secondary improvements are expected in the domain of dyspnea, asthma control and dysfunctional breathing. The MDRT in people with asthma may contribute to better disease management.

## Methods

### Study design

**Figure 1.**
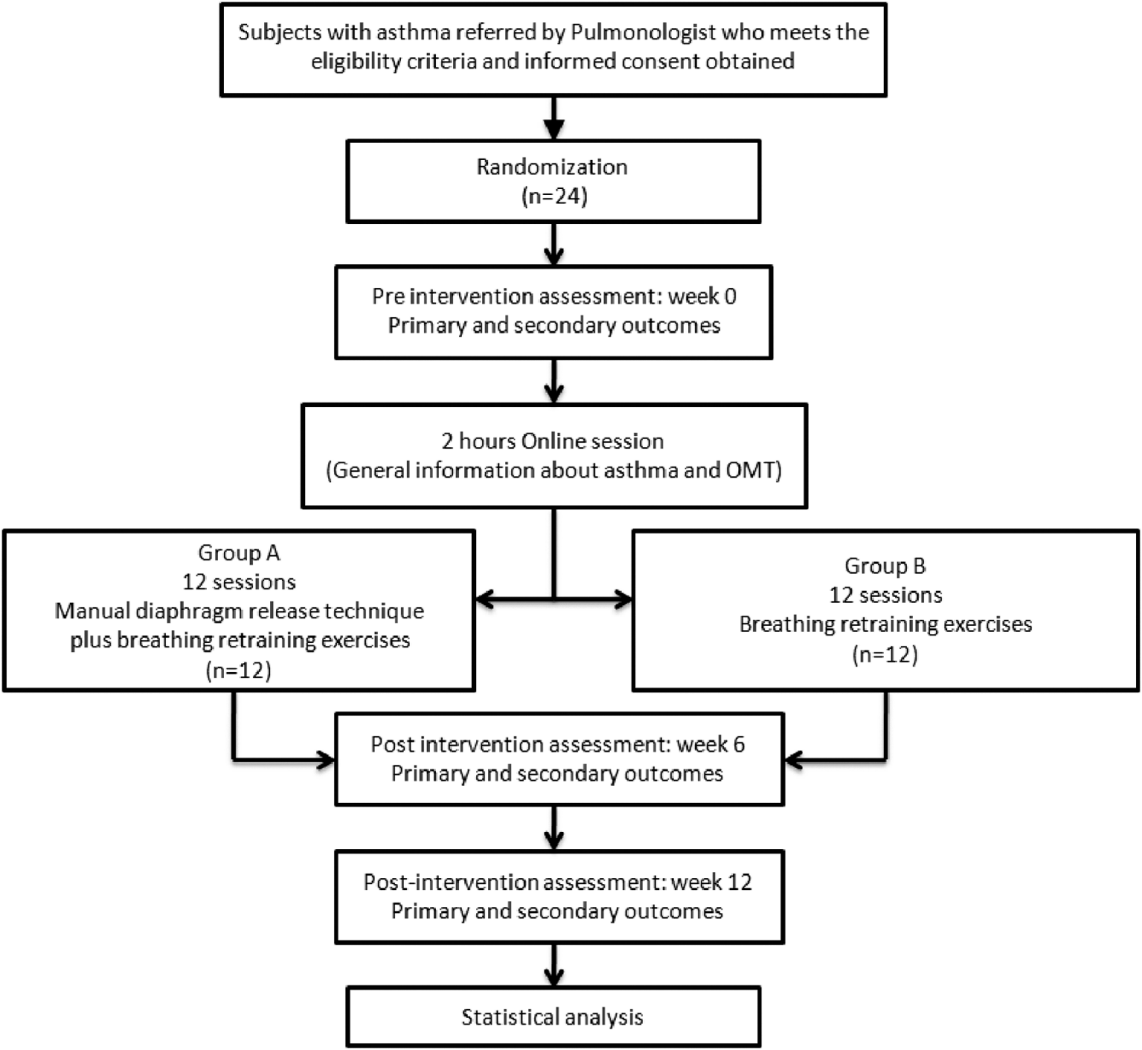
Design of the trial that the flowchart was prepared in accordance with the SPIRIT statement.

The present RCT will be a single centric, two arm parallel equivalence randomized controlled study conducted in accordance with the CONSORT statement. This RCT followed the SPIRIT (Standard Protocol Items: Recommendations for Interventional Trials) statement. ^35^ The completed SPIRIT checklist of the study is uploaded into an approved open repository (Reporting guidelines paragraph). The study’s flowchart is shown in Figure 1.

### Setting

The laboratory of Advanced Physiotherapy at the Physiotherapy Department of the University of West Attica (UNIWA) in Athens will be responsible for coordinating the trial. The recruitment of the patients will be conducted from the Pulmonology Department of the General Oncology Hospital of Kifissia “Agii Anargiri” (GOHK) in Athens (Greece). The hospital’s research committee approved the protocol (4479/09-03-22). Interventions and assessments will take place individually (home-based treatment) in Athens (Greece).

### Recruitment procedures

The participants will be recruited from the GOHK. All participants will be out-patients with diagnosed stable asthma, referred by the director, pulmonologist, of the Pulmonary Department of the GOHK. The same pulmonologist will perform lung function testing on all participants (well-maintained and regularly calibrated equipment), will diagnose asthma and perform the measure of chest expansion. A radiologist will perform the ultrasonography (US) and a statistician will process the required analysis. Two separate physical therapists will implement the DMRT and BRE. All participants will read and sign the consent form. The declaration of consent refers to personal data privacy and participation protection. They will subsequently be informed about a) the objectives, methods, and details of the study, (b) that it will be the volunteers’ decision whether to take part or not, (c) they will always have the right to withdraw from the research even after signing, (d) they can also refuse to answer questions they will not wish or remain in the survey and (e) that the patient’s decision to participate will not affect the provision of our research services.

### Randomization and blinding procedures

Following the initial assessment, eligible volunteers will be randomly assigned to two experimental groups if they meet the inclusion criteria by the secretariat of the Pulmonology Department of the GCHAA. The randomization will be carried out using sealed envelopes. Individuals will have their allocation concealed through the use of sealed envelopes that are sequentially numbered and opaque. ^36^ The envelopes will be opened only by the primary researcher, who is responsible for the research project and coordination of the study. The pulmonologist who will perform lung function testing on all participants (well-maintained and regularly calibrated equipment), diagnose asthma and perform the measure of chest expansion, the radiologist who will perform the ultrasonography (US), and the two separate physical therapists, who will implement the DMRT and BRE will be unaware of the group allocation.

### Participants and eligibility

The pulmonologist of the trial will approach all eligible patients for recruitment into the study. They will be assessed through a set of questions to confirm eligibility. All patients will be requested to provide written informed consent (signed by the main researcher, the subject, and two witnesses) to participate in the study, including permission to publish results, with the understanding that they can withdraw at any time.

### Participants’ characteristics

At least 24 adults will be recruited for the study.

Inclusion criteria: aged 18-60 years, diagnosed with stable asthma ^37^ and correct use of the inhaler technique.

Exclusion criteria: Participation in other physical therapy methods, the presence of cardiopulmonary conditions, prior cardiothoracic or abdominal surgeries, recent chest wall or abdominal trauma, unstable hemodynamic parameters (systolic arterial pressure >140 mmHg and diastolic >90 mmHg), inability to comprehend verbal instructions required for outcome assessments, pregnancy, neurological ailments, and concurrent involvement in interventional programs.

### Initial assessments

A pulmonologist of GOHK with years of experience diagnosing and treating asthma patients will examine the chest expansion, the pulmonary function, and diagnosis of asthma. The radiologist will perform the US assessment, and the secretariat of the GOHK will administer the questionnaires in a random order and collect all data.

**Interventions**

The pulmonologist and the main researcher of the protocol will organize two online sessions with all the participants after the randomization. In one session, participants will be informed by the pulmonologist about (a) general asthma information, (b) asthma triggers, (c) recognition of asthma symptoms, (d) medication and proper use of asthma medications (inhaler techniques), (e) smoking, and (f) asthma control (symptom control/future risk domains/long-term goals). ^38^ As for the last domain (asthma control), information will be provided regarding, (i) the dynamic changes of asthma, (ii) the recognition of these changes based on PEF values and symptoms, (iii) the importance of early detection of clinical signs of worsening and the immediate initiation of appropriate medication.

At the second online session the participants will be informed by the main researcher about (a) the breathing pattern, (b) the pathological pattern of breathing that is developing both in stable phase and in paroxysm, ^39^ (c) the role of physiotherapy and specifically about the BRE in PR, (d) the formation of a trusting relationship between a person living with asthma and a medical professional (e) the importance of self-efficacy in asthma self-management ^40^^–^^43^ and (f) the self-efficacy enhancement process.

Following the two online sessions, there will be a discussion between patients and health professionals (pulmonologist - main researcher) lasting approximately one hour, during which patients can share their objectives, values, apprehensions, support and commendations. The structure of the online session will be determined by a) the long-term objectives for asthma management ^5^ and b) the health belief model. ^44^^,^^45^ Regarding the treatment sessions, every participant will receive twelve sessions twice weekly for six weeks, lasting one hour per session. Individual sessions will be provided in patients’ homes under the same conditions ( *e.g.*, day, time of day, temperature, therapeutic bed, and patient’s position).

Physiotherapy intervention will be provided by two physical therapists, trained by the professor of chest physiotherapy and the professor of manual therapy respectively, at the Physiotherapy Department, UNIWA. Any departure from the administration of treatment sessions (such as missing more than one appointment) or any exacerbation of symptoms will lead to exclusion. Brief descriptions of the interventions planned for each group are provided below.

Intervention Group: This group will receive MDRT plus BRE. MDRT is intended to stretch and mobilize the diaphragmatic muscle fibers indirectly. MDRT will be applied as described by Rocha and his colleagues. ^18^

MDRT: Participants will be instructed to lie in a supine position with relaxed limbs. The therapist will be positioned at the patient’s head. Manual contact will be made using the hypothenar region and the last three fingers bilaterally, placed under the seventh to tenth rib costal cartilages. The therapist’s forearms will align towards the patient’s shoulders. During inhalation, the therapist will apply gentle pulling and lateral elevation of the ribs in the inspiratory phase at the points of contact. As the participant exhales, the therapist’s touch will deepen towards the inner costal margin while maintaining resistance. This connection will further deepen within the costal margin in subsequent respiratory cycles. Regarding respiratory volumes, patients will be instructed to breathe progressively deeper from set to set, aiming for maximal diaphragmatic excursion and stretch. It is essential to ensure that the therapist’s grip on the lower thoracic aperture is maintained throughout. The entire process will consist of two sets of 10 repetitions, separated by a 1-minute interval lasting 10 minutes. ^46^

BRE: BRE will be conducted for 30 minutes. The primary objective of these exercises is to mitigate hyperventilation, hypocapnia, and dysfunctional breathing—common symptoms in individuals with asthma. ^47^ The initial step involves identifying and inhibiting an abnormal upper thoracic respiratory pattern and re-education of diaphragmatic and slow nasal breathing. Additionally, brief respiratory pauses will be introduced after each exhalation. ^48^ Subsequently, BRE aims to integrate the new breathing pattern into daily life. This stage focuses on incorporating diaphragmatic and slow nasal breathing into various daily activities, encompassing physical activities (e.g., speaking, swimming, walking, gardening), social activities (e.g., playing with children or pets), and work-related activities (e.g., managing work stress). ^49^

The phases of BRE will consist of: i) identification of the abnormal breathing pattern, ii) diaphragmatic breathing, ii) nose breathing, iii) slow breathing with controlled breath-holding at the end of exhalation, iv) adaptation of the new breathing pattern in everyday life activities and various positions (supine, semi-sitting, sitting), and v) breathing control in speech. The repetitions and sets of BRE are indicative, given that our priority was the progression and individualization during the sessions. For instance, the first session may only include recognition of the abnormal breathing pattern exercise and practice diaphragmatic and nose breathing retraining exercise. At the beginning of every session, each participant will be assessed for their compliance concerning the exercises (use of a calendar or via questions about their exercise). If a patient has not comprehended or cannot perform the exercises correctly, the previous session will have to be repeated. Once they have fully understood the instructions and execution, they will proceed to the subsequent BRE.

The Table 1 illustrates the Key parameters of the rehabilitation program.

**Table 1.** Key parameters of the rehabilitation program.

Intervention		Patient position	Instructions to participants	Time	Sets/Breathing cycles	Weekly repetition
**MDRT**		- Lie supine with relaxed limbs - 90 degrees flexion of the knees & hips		20-25 min	2S x 10BC	2/Week
**BRE**	**Recognition of the abnormal breathing pattern**	- A pillow between the knees & head. - Half-lying supine (45 degrees of spine flexion from the neutral supine position).	- *“Put one relaxed hand on your upper chest and the other on your stomach and clavicle”* - *“Breathe normally, slowly, in your own manner”* - *“Take a big breath”*	30 min	1S x 5BC	2/Week
	**Practice diaphragmatic and nose breathing retraining**	- Half-lying supine position - Pillows behind the head & knees - one relaxed hand over the stomach and the other on the upper chest and clavicle	- *“Close your mouth and try to breathe easily, slowly and softly through your nose”* - *“Be careful not to inhale with “big” breaths, try during inhalation to raise only the stomach”* - *“Exhalation is passive through the nose and lets your stomach fall gently”*		1S x 5BC	
	**Slow breathing and controlled breath holding after exhalation**	- Half-lying supine position - Pillows behind head and knees - One relaxed hand over stomach and the other on upper chest and clavicle	- *“Close your mouth and try to breathe easily, slowly and softly through your nose”* - *“Relax your shoulder & chest”* - *“Try to slow down your breaths”* - *“Sometimes it helps to count in your mind while you are breathing, e.g. breathe in for a slow count of 2 and breathe out for a slow count of 3until feel empty your stomach”* - *“Keep empty your stomach for 3-5 seconds”*		1S x 10BC	
	**New breathing pattern in everyday life activities and various positions**	- Supine - Semi-sitting - Sitting	- *“Try to breathe in and out slowly and through the nose, even in the most challenging conditions”* - *“You will be able to recognize faulty breathing patterns, and you will be able to correct them”* - *“Try to use a calendar or a mobile phone reminder to implement a diaphragmatic slow nose breathing pattern and breathing exercises”*		3S x 5BC	
	**Breathing control in speech**	- Supine - Semi-sitting - Sitting	- *“You must budget airflow to control the number of words you say per breath and the tone and loudness of particular words in a sentence”* - *“Apply a slow, through the nose, diaphragmatic breathing pattern at speech (max 4-6 words spoken during slow exhalation, with breathing pauses, and slow inhalations)”* - *“Breathe out so that your lungs feel empty, and, before inhaling again”* - *“At yawning and at sighing close your mouth”* - *“Poor voice quality (hoarseness, roughness, or a strained/strident voice) results from inadequate or badly controlled airflow. Speech prosody: rhythm and intonation”*		3S x 5BC	

Control Group: This group will receive only BRE, as mentioned in the intervention group.

### Outcome measures

The evaluations will take place at three time - points: prior to treatment (week 0), post-treatment (week 6), and three months subsequent (week 12). The primary outcomes will entail the measurement of diaphragm excursion using ultrasound ^50^ and chest expansion (CE) using a tape measure. ^51^ Secondary outcomes will also be employed: maximal inspiratory and expiratory pressures (MIP/MEP), ^52^^,^^53^ dysfunctional breathing (Nijmegen questionnaire – - NQ), ^54^ asthma Control (ACT), ^55^^,^^56^ quality of life (SF-12v2), ^57^ and dyspnea (Borg scale). ^58^ Table 2. illustrates the SPIRIT ^34^ schematic protocol of the study along with the schedule of assessments.

**Table 2.** Standard protocol items: recommendations for interventional trials of this study (SPIRIT).

TIME POINT	Enrollment (-T1)	Allocation (T0)	Pre-intervention (T1)	Intervention	Post-Intervention	
					(T2)	(T3)
ENROLLMENT:						
Eligibility screen	X					
Informed consent	X					
Demographic information	X					
Allocation		X				
Intervention						
MDRT + BRE				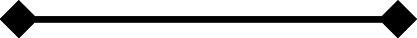		
BRE				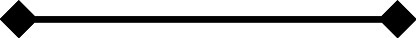		
ASSESMENTS			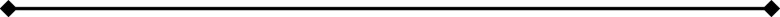			
Neurological and cardiovascular assessments	X					
Primary outcomes: US			X		X	X
CE			X		X	X
Secondary outcomes: MIP/MEP			X		X	X
NQ			X		X	X
ACT			X		X	X
Borg scale			X		X	X
SF-12v2			X		X	X

-T1: one month before first treatment; T0: one week before first treatment (week 0); T1: 20 minutes before first treatment; T2: after the last treatment, (week 6); T3: follow-up 6 weeks from the last treatment (week 12).

***Primary outcome measures***

**Diaphragmatic excursion assessment with ultrasonography:** Numerous studies have confirmed the effectiveness of the US to evaluate diaphragmatic function. Since 1970, ^59^ the US has been utilized to assess the diaphragm’s mobility. Modern medicine accepts its use as a completely safe method. The diagnostic US has 93% sensitivity and 100% specificity. ^17^ The examiners are not exposed to any radiation, and there is no need for special preparation, allowing for as many safe applications as required. The US offers the option of a dynamic inspection of the affected area in real time. The diaphragmatic excursion is measured in cm/mm. In this study the excursion of the diaphragm will be measured using the ultrasound device, E-CUBE 8LE (Seoul, Republic of Korea).

**Chest wall expansion (CWE):** In clinical and research practice, the inch tape measure is an alternative method for assessing chest expansion. ^60^ By placing the tape measure at the level of the axilla (about the level of the sternal angle of Louis), the level of the xiphoid process, or between the xiphoid process and the umbilicus, the therapist identifies the upper, middle, and lower chest wall expansion, respectively. The therapist should repeat the measurement at least three times for each level for higher fidelity. ^51^ A tape measure (in centimeters) will assess the variance between values recorded during deep inhalation and exhalation, with greater values signifying improved results. ^51^ Norms have been developed according to age and sex. ^60^

***Secondary outcome measures***

**Nijmegen Questionnaire (NQ):** The Nijmegen Questionnaire is a reliable and valid tool for assessing dysfunctional breathing ^62^ in clinical practice and research. It is designed to identify the Hyperventilation Syndrome (HS). A score greater than 23 indicates the presence of HS in the general population. The NQ has shown 91% sensitivity and 95% specificity. ^61^^,^^62^ In previous research, NQ scores of 20, 22 and 23 have been used as cut-off scores to detect HS in subjects with and without asthma. ^63^^–^^65^ The NQ questionnaire has been validated in Greek adults with asthma, providing evidence of validity and reliability of measurements with a cut-off score of >17. ^66^

**Asthma control test (ACT):** The ACT questionnaire is a valid and reliable clinical and research tool. ^67^ Its quick completion time is one of its key features. It has five items, all about the most recent four weeks. ^68^^,^^69^ The ACT evaluates the frequency of wheezing and other general asthma symptoms and the need for emergency control self-assessment. The score ranges from 5 (poor control of asthma) to 25 (good control of asthma). An ACT score >19 indicates controlled asthma. The ACT has been validated in the Greek population with asthma and has shown high indices of internal consistency (0.72) and test–retest reliability (IR = 0.85). ^59^

**Sf-12v2 questionnaire:** A simplified version of the SF-36 includes medications, and how well asthma affects daily functioning and overall asthma. The SF-12v2 is a practical, reliable, and valid way to assess physical and mental health. With one or two questions per domain, it assesses the exact eight health dimensions as the SF-36v2, which includes Physical Functioning, Role-Physical, Bodily Pain, General Health, Vitality, Social Functioning, Role-Emotional, and Mental Health. The SF-12 is a valid alternative to the lengthy SF-36 for the self-assessment of quality of life by assessing the health status of healthy and patient population groups. ^69^ Higher ratings indicate better physical and mental wellbeing, ranging from 0 to 100. ^70^ It has been suggested that a cut-off of 50 or less can be used to identify a physical condition, while a score of 42 or less may signify clinical depression. ^70^ The Sf-12v2 has been weighted in the Greek general population. ^71^

**Borg scale:** The Borg dyspnea scale is a simple, non-proprietary scoring system. It is extensively used in clinical and research practice to evaluate symptoms of shortness of breath and provides valuable data. ^72^^,^^73^ It begins with 0 (no dyspnea), and goes up to 10 (extreme dyspnea). As a result, healthcare professionals must give patients enough time to learn and ensure they comprehend it before using it. ^74^

### Anticipated dates of trial commencement and completion

The study started in January 2023 and is scheduled to be completed in January 2024.

### Sample size calculation

The sample size calculation was based on an alpha (α) level of 0.01, a power (β) of 0.85, and a large effect size, Cohen’s d, of 1.86 for a two-tailed independent t-test. This effect size was calculated based on the results reported by Rocha et al. (2015), ^18^ specifically from the mean (SD) values of the control and experimental groups following six manual therapy intervention sessions. Furthermore, the calculation accounted for an anticipated attrition rate of approximately 15%, foreseeing the potential for participant dropout or exclusion. This comprehensive approach to sample size calculation ensures the robustness and reliability of the study’s findings, enabling a precise detection of the large effect size with high statistical confidence. This resulted in a total sample size of 24 people (12 per group) considering a withdrawal rate of 15%. ^75^

**Data collection methods**

Data regarding the outcomes pre and post-intervention will be meticulously collected from the main researcher. The collected information will undergo a thorough examination to analyze the outcomes. Throughout the study, the professor of chest physiotherapy (supervisor) will closely oversee the main researcher administering the study’s data. To ensure the patient adheres to the study, timely messages will be sent to their mobile phones, providing information and reminders regarding upcoming sessions. A 10% drop-out rate has been considered in the sample size calculation, thus minimizing potential interference with the study results.

**Data management**

Evaluation data will be sourced from a predefined spreadsheet containing baseline characteristics. A secure database will be employed to store all research-related data securely. Paper copies of evaluation forms signed informed consent forms, and other non-electronic documents will be securely stored in the study environment. A comprehensive backup of the data entries will be generated once a month until the conclusion of the trial.

Upon completion of the study, the Excel spreadsheet will be published and forwarded to the statistician for the necessary analysis. A checklist will help prevent data loss from inefficient staff procedures. Given the extensive follow-up assessment for this experiment, participant retention and completion of follow-up assessments will be notably high. After six weeks from the end of interventions, the participants will be invited to a follow-up examination (12 weeks from the beginning of the trial).

### Statistical analysis

The investigation will employ IBM SPSS software, specifically version 28, as the primary analytical tool for comprehensive data processing. Descriptive statistics will be employed to elucidate the distributional characteristics of the dataset. Subsequently, the Shapiro-Wilk test will be utilized to assess data normality for each dependent variable (including US, CWE, ACT, NQ, SF-12v2, and Borg scale) independently. Should normality assumptions be met, parametric tests, specifically the 2×3 ANOVA repeated measures, will be employed, accompanied by Bonferroni adjustment, to investigate the interaction between intervention (experimental and control groups) and time points (0, 6, and 12 weeks). Moreover, to evaluate the homogeneity of variance, Levene’s test will be conducted. The predetermined level of statistical significance is set at p<0.05.

**Monitoring**

***Data monitoring***

A data monitoring committee of members from the UNIWA Physiotherapy Department will periodically review the accumulating data, determining if the trial should be modified or discontinued.

***Harms***

A clinical staff is going to supervise the entire procedure. Any adverse events will be immediately reported throughout the trial to the Physiotherapy Department of the UNIWA committee.

***Auditing***

An evaluation of the experiment will be performed each month. Every deviation from the protocol will be recorded and addressed.

**Ethics and dissemination**

***Research ethics approval***

The Ethics Committee of the UNIWA in Greece approved this study under protocol 90853/04-10-2022. The study follows the Helsinki Declaration’s “Ethical Principles of Medical Research Involving Human Subjects.” Protocol modifications will be disclosed to the Ethics Committee as soon as possible. The trial has been proactively registered in the ClinicalTrials.gov database with the identification number NCT05709054.

***Protocol amendments***

The study has already been modified and accepted in accordance with the Ethics Committee of the UNIWA in Greece suggestions. As a result, further modifications could not be made.

**Consent**

Study participants will receive a translated version of the study protocol. Prior to participation, all individuals involved in the study will be fully informed, and written consent will be obtained from each participant.

### Confidentiality

The main researcher will collect personal data during the trial. A confidentiality statement on the permission form will be signed by the main researcher, the subject, and two witnesses. Whenever necessary to disclose information for the study, consent from the patients will always be obtained with utmost assurance of confidentiality.

### Access to data

The main researcher and the statistician will have access to the final trial datasheet.

### Ancillary and post-trial care

The entire procedure will be conducted under the oversight of clinicians and the physiotherapy department of the UNIWA committee. After the trial sessions, the participants will be under supervision for about four weeks so that they can take care if there is any harm.

## Discussion

Although several studies assessing the efficacy of MT in obstructive lung diseases (OLD) have been conducted, drawing definitive conclusions is challenging due to their conflicting results. ^45^ A recent systematic review (SR) of the existing literature aimed to identify indications that may underscore the need for differentiated manual therapy (MT) approaches targeting the zone of apposition (ZOA) of the diaphragm in individuals with OLD suffering from pathological adaptations of their chest wall or from respiratory symptoms. ^76^ This SR outlined a model illustrating how MT delays the onset of fatigue in respiratory muscles. Additionally, it demonstrated that there is no evidence supporting the effectiveness of MT on the diaphragm for treating individuals (adults) with asthma. ^76^ It is noteworthy that two previous studies have examined how MT on the ZOA of the diaphragm hampers the diaphragm’s function and pulmonary function in childhood asthma (RCT) ^33^ and in adults with asthma ^24^ (pilot study) accordingly. Up to this point, no RCT has comprehensively examined the impact of MDRT, in particular, on the ZOA by MDRT of the diaphragm in adults suffering from asthma.

The primary objective of this RCT is to address the existing literature gap concerning the impact of MDRT on enhancing the functional parameters of individuals with asthma, thereby indicating the need for further research in this domain. Additionally, considering the prevalent pathological changes in the diaphragm muscle among individuals with asthma, it is crucial to determine which anatomical regions and hands-on therapy techniques are most effective for increasing diaphragm excursion in adults with asthma.

The protocol of this study is designed in accordance with accepted practices concerning randomization, concealed allocation, blinding of examiners, and appropriate sample size calculation. This rigorous approach ensures the reliability and robustness of the study’s findings. What sets this study apart is its original investigation into the mechanism of the MDRT on the ZOA of the diaphragm in individuals living with asthma. Finally, several significant constraints must be considered. The study sample is drawn exclusively from a single hospital, potentially limiting the generalizability of the findings. Additionally, the extended experimental period and the substantial number of sessions may lead to participant dropouts.

### Study status

The study started in January 2023.

### Study dissemination

This work will be presented in International Conference Proceedings and published in an indexed journal.

## Conclusions

This RCT will be innovative as it will, for the first time, provide evidence of the effect of the MDRT on diaphragm function in people with asthma. MT is a low-cost alternative and supplementary therapy to standard treatment procedures that might improve the biomechanics of respiration in pulmonary rehabilitation.

## Data availability

### Underlying data

No data are associated with this article.

### Reporting guidelines

Figshare: SPIRIT checklist for ‘The effect of manual therapy on diaphragm function in adults with asthma: Protocol for a randomized controlled trial’, https://doi.org/10.6084/m9.figshare.24191106. ^77^
