# Supplementary material for: The effect of manual therapy on diaphragm function in adults with asthma: Protocol for a randomized controlled trial
Source: F1000Res. 2024 Mar 19;12:1361. Originally published 2023 Oct 18. [Version 2] doi: 10.12688/f1000research.141455.2 (PMC11445601; doi:10.12688/f1000research.141455.2)
Supplement: Peer review history

# Reviewer report: Reviewer response for version 2

**DOI:** 10.5256/f1000research.163150.r257334

**Published:** 2024-04-25

**Authors:** Ragab Elnaggar

**Affiliations:** 1 Department of Physical Therapy for Pediatrics, Faculty of Physical Therapy, Cairo University, Giza, Egypt; 2 Department of Physical Therapy and Health Rehabilitation, College of Applied Medical Sciences, Prince Sattam Bin Abdulaziz University, Al Kharj, Riyadh Province, Saudi Arabia

I appreciate the authors' thorough efforts in addressing the concerns raised in the previous review round. Their revisions have significantly improved the manuscript.

Is the study design appropriate for the research question?

Partly

Is the rationale for, and objectives of, the study clearly described?

Partly

Are sufficient details of the methods provided to allow replication by others?

Partly

Are the datasets clearly presented in a useable and accessible format?

Not applicable

Reviewer Expertise:

Physical Therapy

I confirm that I have read this submission and believe that I have an appropriate level of expertise to confirm that it is of an acceptable scientific standard.

# Reviewer report: Reviewer response for version 2

**DOI:** 10.5256/f1000research.163150.r257335

**Published:** 2024-04-04

**Authors:** Luis Vicente Franco Oliveira

**Affiliations:** 1 Human Movement and Rehabilitation Post Graduation Program, Evangelical University of Goiás, Anápolis, Goiás, Brazil

I am satisfied with the changes made by the authors. All suggestions were accepted.

 I declare that I have no conflict of interest with the manuscript or the authors.

Is the study design appropriate for the research question?

Yes

Is the rationale for, and objectives of, the study clearly described?

Yes

Are sufficient details of the methods provided to allow replication by others?

Yes

Are the datasets clearly presented in a useable and accessible format?

Not applicable

Reviewer Expertise:

PhD in Health Sciences, specialist in Respiratory Physiotherapy and Pulmonary Rehabilitation and Cardiorespiratory Sleep Disorders.

I confirm that I have read this submission and believe that I have an appropriate level of expertise to confirm that it is of an acceptable scientific standard.

# Reviewer report: Reviewer response for version 1

**DOI:** 10.5256/f1000research.154901.r223569

**Published:** 2024-02-20

**Authors:** Ragab Elnaggar

**Affiliations:** 1 Department of Physical Therapy for Pediatrics, Faculty of Physical Therapy, Cairo University, Giza, Egypt; 2 Department of Physical Therapy and Health Rehabilitation, College of Applied Medical Sciences, Prince Sattam Bin Abdulaziz University, Al Kharj, Riyadh Province, Saudi Arabia

This protocol for a prospective, randomized clinical trial will assess the effect of manual diaphragm release technique (MDRT) on diaphragm function in individuals with asthma. While, based on a brief search of the database, previous evidence on the effect of this intervention is likely scarce, there is, therefore, a need for more well-controlled trials, employing adequate methodology, to conclusively evaluate the role of MDRT for patients with asthma.

- I partially disagree with the assertion that “The proposed protocol is the first to examine the effectiveness of MRDT on diaphragm function in individuals with asthma”. There is a previous publication on the role of MRDT in pediatric patients with asthma (1).

- Asthma encompasses heterogeneous phenotypes that vary widely in terms of their underlying causes, triggers, clinical manifestations, and response to treatment. Furthermore, the severity of asthma can range from mild and intermittent symptoms to severe and persistent symptoms that require frequent medical intervention. Understanding the heterogeneity of asthma is important because it helps healthcare professionals tailor treatment approaches to individual patients. It is unclear in this protocol which type/severity of asthma would be considered.

- While reading the introduction section, I noticed that it lacks a comprehensive background on patients with asthma, their diaphragmatic function, and the potential benefits of MDRT in addressing this issue. I believe that including these key elements would greatly enhance the clarity and contextual understanding of the intended study. Providing a brief overview of the impact of asthma on diaphragmatic function, respiratory muscle strength, or altered breathing patterns, and emphasizing the potential of MDRT as a therapeutic intervention would help readers grasp the significance of the study and its potential implications for asthma management. I encourage the authors to consider revising the introduction accordingly to provide a more comprehensive background and set a firm foundation for the remainder of the study.

- The significance of the study is not clear. Authors may need to justify the importance of their work, highlight the impact it has on the research field, and define its contribution to new knowledge.

- More importantly, they need to pinpoint the research gap (in light of the related literature) and make it clear what drove them to conduct the study.

- Again, the statement “As for asthma, there is no data for the efficacy of diaphragm MT methods, except for one pilot study” is not true and should be revised in light of the aforementioned publication.

- I have a concern regarding the study power. A sample size of 24 is a considerably small sample. The authors conducted an a priori power analysis to determine the minimum sample size necessary for detecting clinically significant results. They employed a substantial effect size (d = 1.36) and the minimum acceptable power level (80%) in their analysis. However, this approach may present challenges in detecting moderate or small changes in the outcome variable, potentially resulting in inconclusive findings and limiting the interpretability of the study conclusions.

- The authors aim to recruit a minimum of 24 participants, encompassing a broad age range from 8 to 60 years. However, it is recommended that the authors consider prioritizing either pediatric or adult patients for a more focused investigation.

- It is crucial to incorporate a thorough and detailed description of the pulmonary rehabilitation program within this protocol, as its current absence hinders the comprehensive understanding of the intervention being studied.

- The data analysis plan is appropriate. The utilization of 2x3 repeated measures ANOVA with Bonferroni adjustment will be used to examine the interaction between intervention offer advantages for allowing for the examination of within-subject changes across multiple conditions or time points and accounting for the correlated nature of data within subjects.

- In summary, the implementation of the study as outlined in this protocol has the potential to offer supplementary or corroborating evidence concerning the efficacy of MDRT in individuals diagnosed with asthma.

Is the study design appropriate for the research question?

Partly

Is the rationale for, and objectives of, the study clearly described?

Partly

Are sufficient details of the methods provided to allow replication by others?

Partly

Are the datasets clearly presented in a useable and accessible format?

Not applicable

Reviewer Expertise:

Physical Therapy

I confirm that I have read this submission and believe that I have an appropriate level of expertise to confirm that it is of an acceptable scientific standard, however I have significant reservations, as outlined above.

## References

[1] : Prospective Effects of Manual Diaphragmatic Release and Thoracic Lymphatic Pumping in Childhood Asthma. *Respir Care* .2019;64(11) : 10.4187/respcare.06716 1422-1432 10.4187/respcare.06716 31337743

# Response

**Published:** 2024-02-20

**Authors:** DIMITRIOS TSIMOURIS

**Affiliations:** Department of Physiotherapy, Not Applicable, Athens, Greece

Dear Ragab Elnaggar,

 Thank you for your thorough review and insightful feedback on our study protocol. Your contributions have significantly enhanced the quality of our work. We will meticulously address all the concerns raised and further refine our manuscript.

 Yours sincerely,

 Mr Dimitrios Tsimouris

 --

 PhD candidate

 Department of Physiotherapy

 University of West Attica

 Athens, Greece

# Response

**Published:** 2024-02-26

**Authors:** DIMITRIOS TSIMOURIS

**Affiliations:** Department of Physiotherapy, Not Applicable, Athens, Greece

**Dear Prof. Ragab Elnaggar,**

 We would like to thank you for reviewing our manuscript and giving us the opportunity to revise it. We appreciate the time and effort you put into this to further improve our work.

 Before presenting our responses to your comments, we deemed it important to clarify that our study will only focus on adults with mild to moderate severity of asthma. Unfortunately, we found a typographical error in the inclusion criteria of our study, where "18" was mistakenly written as "8." We acknowledge that this significantly affected your assessment. Given the above, we here by provide our responses in the same order as you presented your comments. Please find our replies below:

**Comment 1: « *I partially disagree with the assertion that “The proposed protocol is the first to examine the effectiveness of MRDT on diaphragm function in individuals with asthma”. There is a previous publication on the role of MRDT in pediatric patients with asthma (1).»***

*Thank you for this comment. We agree that this part of the manuscript was a bit overstated. Following your suggestion,* *we decided to delete the sentence to improve the clarity of this section.*

**Comment 2: « *While reading the introduction section, I noticed that it lacks a comprehensive background on patients with asthma, their diaphragmatic function, and the potential benefits of MDRT in addressing this issue. I believe including these key elements would greatly enhance the clarity and contextual understanding of the intended study. Providing a brief overview of the impact of asthma on diaphragmatic function, respiratory muscle strength, or altered breathing patterns and emphasizing the potential of MDRT as a therapeutic intervention would help readers grasp the significance of the study and its potential implications for asthma management. I encourage the authors to consider revising the introduction accordingly to provide a more comprehensive background and set a firm foundation for the remainder of the study.»***

*Thank you for this comment. We agree that this part of the manuscript was inadequate. Following your suggestion, we have modified the introduction, providing a more comprehensive background.* We have added the text as follows:

*« It's also important to note that the mechanical disadvantage of the diaphragm in asthma can result in an increased workload for all inspiratory muscles, particularly during exercise, where dynamic hyperinflation may occur, leading to heightened dyspnea ^15^* *^,16^»*

 « Finally, although specific diaphragm MT techniques have not been documented yet, recent studies have reported evidence for their positive effect on pulmonary rehabilitation (PR). ^25 ^In particular, the manual diaphragm release technique (MDRT) aims to directly stretch the muscle fibers of the diaphragm, as detailed in Rochas’ research. ^18 ^The study showed an improvement in diaphragm’s mobility, maximum inspiratory pressure (MIP), and exercise capacity (EC) in people suffering from COPD. ^18^ We deem it pertinent to mention that previous studies have demonstrated that even a single MT session can have a positive effect on chest wall mechanics, dyspnea, and peripheral oxygen saturation (SpO2) in individuals with COPD. ^26, 27^ *Therefore, dyspnea, being one of the primary symptoms of asthma, can adversely affect both exercise capacity (EC) levels and overall quality of life (QoL). ^28-31^* According to a study ^26^ a single MT session of soft tissue and joint mobilization immediately improved dyspnea (Borg Scale 0-10, pre: 2.3 ± 0.8 vs 1.8 ± 0.5). The authors reported that the mechanism underlying this improvement could be the increase in respiratory muscle length and thoracic cage flexibility induced by MT, consequently reducing breathing effort and the development of dyspnea in individuals with COPD. ^32^ As for asthma, there is currently no data regarding the efficacy of diaphragm MT methods, except for the pilot study conducted by Macias and colleagues ^24^, and the study by Elnaggar and colleagues ^33^, which investigated the efficacy of MDRT in children.

Considering the growing clinical interest in asthma and the recent publications in the field, we believe that a randomized controlled trial (RCT) targeting intervention on the zone of apposition of the diaphragm using the MDRT in adults with asthma for outcomes assessment is warranted.

*.»*

**Comment 3: « *The significance of the study is not clear. Authors may need to justify the importance of their work, highlight the impact it has on the research field, and define its contribution to new knowledge.»***

**Comment 4:** **« *More importantly, they need to pinpoint the research gap (in light of the related literature) and make it clear what drove them to conduct the study.»***

 Thank you for your suggestions. As requested, we highlighted the impact on the research field and the research gap at the discussion session. The text has been correspondingly modified as follows:

“Although several studies assessing the efficacy of MT in obstructive lung diseases (OLD) have been conducted, drawing definitive conclusions is challenging due to their conflicting results. ^45 ^A recent systematic review (SR) of the existing literature aimed to identify indications that may underscore the need for differentiated manual therapy (MT) approaches targeting the zone of apposition (ZOA) of the diaphragm in individuals with OLD suffering from pathological adaptations of their chest wall or from respiratory symptoms. ^76 ^This SR outlined a model illustrating how MT delays the onset of fatigue in respiratory muscles. Additionally, it demonstrated that there is no evidence supporting the effectiveness of MT on the diaphragm for treating individuals (adults) with asthma. ^76 ^It is noteworthy that two previous studies have examined how MT on the ZOA of the diaphragm hampers the diaphragm's function and pulmonary function in childhood asthma (RCT) ^33^ and in adults with asthma ^24^ (pilot study) accordingly. Up to this point, no RCT has comprehensively examined the impact of MDRT, in particular, on the ZOA of the diaphragm by MDRT in adults suffering from asthma.

The primary objective of this RCT is to address the existing literature gap concerning the impact of MDRT on enhancing the functional parameters of individuals with asthma, thereby indicating the need for further research in this domain. Additionally, considering the prevalent pathological changes in the diaphragm muscle among individuals with asthma, it is crucial to determine which anatomical regions and hands-on therapy techniques are most effective for increasing diaphragm excursion in adults with asthma.

The primary objective of this RCT is to address the existing literature gap concerning the impact of MDRT on enhancing the functional parameters of individuals with asthma, thereby indicating the need for further research in this domain. Additionally, considering the prevalent pathological changes in the diaphragm muscle among individuals with asthma, it is crucial to determine which anatomical regions and hands-on therapy techniques are most effective for increasing diaphragm excursion in adults with asthma.

The protocol of this study is designed in accordance with accepted practices concerning randomization, concealed allocation, blinding of examiners, and appropriate sample size calculation. This rigorous approach ensures the reliability and robustness of the study's findings. What sets this study apart is its original investigation into the mechanism of the MDRT on the ZOA of the diaphragm in individuals living with asthma. Finally, several significant constraints must be considered. The study sample is drawn exclusively from a single hospital, potentially limiting the generalizability of the findings. Additionally, the extended experimental period and the substantial number of sessions may lead to participant dropouts.

”

**Comment 5: *«Again, the statement “As for asthma, there is no data for the efficacy of diaphragm MT methods, except for one pilot study” is not true and should be revised in light of the aforementioned publication.»***

 We believe that this comment has been addressed based on our previous replies. After clarifying that there was confusion due to the typographical error, we believe that this statement is now correct. Please do not hesitate to reach out for any further clarification.

**Comment 6: « *I have a concern regarding the study power. A sample size of 24 is a considerably small sample. The authors conducted an a priori power analysis to determine the minimum sample size necessary for detecting clinically significant results. They employed a substantial effect size (d = 1.36) and the minimum acceptable power level (80%) in their analysis. However, this approach may present challenges in detecting moderate or small changes in the outcome variable, potentially resulting in inconclusive findings and limiting the interpretability of the study conclusions.»***

 Thank you for your comment. We understand your concerns about the sample study calculation, and we would like to explain the rationale behind the participant number determined by our power analysis. In 2023, we conducted a systematic review ( *Is manual therapy of the diaphragm effective for people with Obstructive Lung Diseases? A Systematic Review, Respir Med Res. 2023 Jun:83:101002. doi: 10.1016/j.resmer.2023.101002. Epub 2023 Feb 15.)* investigating the effects of specialized mobilization techniques applied to the zone of apposition of the diaphragm in patients with obstructive respiratory diseases, including asthma and COPD. The results of this study revealed a literature gap indicating that the applied techniques on the ZOA of the diaphragm have not been applied in people with asthma beyond, of course, your study (in the pediatric population) and that of Macias et al. 2018 ( *Effects of manual therapy on the diaphragm in asthmatic patients: A randomized pilot studyhttps://doi.org/10.1016/j.ijosm.2018.07.006*). The two studies included in our research represented the closest in design to our own protocol (participants, manual therapy techniques of the diaphragm, diaphragm excursion measured by the US), thus necessitating the power analysis calculation based on them. For your convenience, we hereby provide the two studies in our research, which served as the basis for our study power.

- *Rocha T, Souza H, Brandão DC, et al.: The Manual Diaphragm Release Technique improves diaphragmatic mobility, inspiratory capacity and exercise capacity in people with chronic obstructive pulmonary disease: a randomised trial.* *J. Physiother.* *2015;61(4):182–189. 26386894 10.1016/j.jphys.2015.08.009*
- *Nair A, Alaparthi GK, Krishnan S, et al.: Comparison of Diaphragmatic Stretch Technique and Manual Diaphragm Release Technique on Diaphragmatic Excursion in Chronic Obstructive Pulmonary Disease: A Randomized Crossover Trial.* *Pulm. Med.* *2019;2019:1–7. 30719351 10.1155/2019/6364376 PMC6335861*

These studies closely aligned with the design of our protocol and thus informed our power analysis calculation. The sample size calculation was based on an alpha (α) level of 0.01, a power (β) of 0.85, and a large effect size, Cohen's d, of 1.86 for a two-tailed independent t-test. This effect size was calculated based on the results reported by Rocha et al. (2015), specifically from the mean (SD) values of the control and experimental groups following six manual therapy intervention sessions. Furthermore, the calculation accounted for an anticipated attrition rate of approximately 15%, foreseeing the potential for participant dropout or exclusion. This comprehensive approach to sample size calculation ensures the robustness and reliability of the study's findings, enabling precise detection of the large effect size with high statistical confidence. This resulted in a total sample size of 24 people (12 per group), considering a withdrawal rate of 15%.

**Comment 7: « *The authors aim to recruit a minimum of 24 participants, encompassing a broad age range from 8 to 60 years. However, it is recommended that the authors consider prioritizing either pediatric or adult patients for a more focused investigation.»***

 We agree that this is a mistake. Thank you for pointing this out. We have modified the text according to your comment.

**Comment 8: «** ***It is crucial to incorporate a thorough and detailed description of the pulmonary rehabilitation program within this protocol, as its current absence hinders the comprehensive understanding of the intervention being studied.»***

 Thank you for prompting us to share the treatment regimen with our readers. It was an oversight, not to mention it in detail. The following paragraphs and Table 1. (Key parameters of the rehabilitation program) have been added to the manuscript (Interventions Section):

“Regarding respiratory volumes, patients will be instructed to breathe progressively deeper from set to set, aiming for maximal diaphragmatic excursion and stretch. It is essential to ensure that the therapist's grip on the lower thoracic aperture is maintained throughout.”

“The phases of BRE will consist of i) identification of the abnormal breathing pattern, ii) diaphragmatic breathing, ii) nose breathing, iii) slow breathing with controlled breath-holding at the end of exhalation, iv) adaptation of the new breathing pattern in everyday life activities and various positions (supine, semi-sitting, sitting), and v) breathing control in speech. The repetitions and sets of BRE are indicative, given that our priority was progression and individualization during the sessions. For instance, the first session may only include recognition of the abnormal breathing pattern exercise and practice diaphragmatic and nose breathing retraining exercise. At the beginning of every session, each participant will be assessed for their compliance concerning the exercises (use of a calendar or via questions about their exercise). If a patient has not comprehended or cannot perform the exercises correctly, the previous session will have to be repeated. Once they have fully understood the instructions and execution, they will proceed to the subsequent BRE.”

**Comment 9: « *The data analysis plan is appropriate. The utilization of 2x3 repeated measures ANOVA with Bonferroni adjustment will be used to examine the interaction between intervention offer advantages for allowing for the examination of within-subject changes across multiple conditions or time points and accounting for the correlated nature of data within subjects.»***

*Thank you for reviewing and confirming that our statistical approach is appropriate.*

# Reviewer report: Reviewer response for version 1

**DOI:** 10.5256/f1000research.154901.r216768

**Published:** 2023-10-25

**Authors:** Luis Vicente Franco Oliveira

**Affiliations:** 1 Human Movement and Rehabilitation Post Graduation Program, Evangelical University of Goiás, Anápolis, Goiás, Brazil

This RCT will be innovative as it will, for the first time, provide evidence of the effect of the manual diaphragm release technique on diaphragm function in people with asthma. Manual Therapy is a low-cost alternative and supplementary therapy to standard treatment procedures that might improve the biomechanics of breathing in pulmonary rehabilitation.

 Studies aimed at investigating intervention strategies that seek to improve the functioning of the main muscle of our breathing (diaphragm) are extremely important around the world. The authors propose a low-cost intervention strategy without any risk to patients. I believe that the results of this study protocol will make important contributions to Pulmonology and Respiratory Physiotherapy around the world. Below I describe some suggestions to improve the presentation of the study protocol.

 The title of the study complies with the PICOS strategy, characterizing the proposal well. It is noteworthy that the study protocol is already registered at Clinical Trials.gov (ID: NCT05709054). The manuscript contains all sessions necessary for a study protocol. The abstract of the manuscript is well written and well designed, summarizing the study proposal very well.

- I suggest stating in the title of Figure 1. Design of the trial that the flowchart was prepared in accordance with the SPIRIT statement.
- In the “Methods - Study design” section I suggest maintaining what is described about the SPIRIT (Standard Protocol Items: Recommendations for Interventional Trials) statement, in relation to the protocol design, however adding that the study will be a randomized controlled study conducted in accordance with the CONSORT statement.
- I suggest adding the acronym SPIRIT to the end of the title of Table 1.
- In the primary outcome assessment item, add the commercial references of the ultrasound equipment that will be used to analyze the movement of the diaphragm. These details are important to allow other researchers to reproduce the study.
- I suggest improving the description of the statistical analysis, adding more details about the analyses. In this same item, please add the commercial description of the software to be used.
- The study does not mention whether there are conflicts of interest between the authors. It would be interesting to mention if there is any type of conflict.
- I suggest that the authors standardize bibliographic references. There are some non-standard ones.

I would also like to congratulate the authors for the brilliant proposal.

Is the study design appropriate for the research question?

Yes

Is the rationale for, and objectives of, the study clearly described?

Yes

Are sufficient details of the methods provided to allow replication by others?

Yes

Are the datasets clearly presented in a useable and accessible format?

Not applicable

Reviewer Expertise:

PhD in Health Sciences, specialist in Respiratory Physiotherapy and Pulmonary Rehabilitation and Cardiorespiratory Sleep Disorders.

I confirm that I have read this submission and believe that I have an appropriate level of expertise to confirm that it is of an acceptable scientific standard, however I have significant reservations, as outlined above.

# Response

**Published:** 2023-10-26

**Authors:** DIMITRIOS TSIMOURIS

**Affiliations:** Department of Physiotherapy, Not Applicable, Athens, Greece

Dear Luis Vicente Franco Oliveira,

 I extend my sincere thanks for your meticulous evaluation of our study protocol and for providing valuable feedback that helped us improve our work's quality. Please be reassured that we will address all the issues mentioned and further improve the manuscript.

 We are excited to share our findings with the broader research community and hope they interest readers. The publication of our study will provide new insights and stimulate further research in this area.

 Yours sincerely,

 Mr Dimitrios Tsimouris

 --

 PhD candidate

 Department of Physiotherapy

 University of West Attica

 Athens, Greece

# Response

**Published:** 2024-02-26

**Authors:** DIMITRIOS TSIMOURIS

**Affiliations:** Department of Physiotherapy, Not Applicable, Athens, Greece

**Dear Prof. Luis Vicente Franco Oliveira,**

 We would like to thank you for allowing us to revise the manuscript. We appreciate the time and effort you put into this to improve our work further. We have responded to all the comments. Please find our replies below.

**Comment 1:** ***«I suggest stating in the title of Figure 1. Design of the trial that the flowchart was prepared in accordance with the SPIRIT statement.»***

 Thank you for the suggestion. The heading of Figure 1 has been changed.

**Comment 2:** ***«In the “Methods - Study design” section, I suggest maintaining what is described about the SPIRIT (Standard Protocol Items: Recommendations for Interventional Trials) statement in relation to the protocol design, however adding that the study will be a randomized controlled study conducted in accordance with the CONSORT statement»***

 Thank you for your comment. We apologize for the mistake you pointed out. We have now added that our study is a randomized controlled study conducted in accordance with the CONSORT statement.

**Comment 3:** ***«I suggest adding the acronym SPIRIT to the end of the title of Table 1»***

 Thank you for this suggestion. The acronym SPIRIT has been added to the end of the title of Table 1, as you suggested.

**Comment 4:** ***«In the primary outcome assessment item, add the commercial references of the ultrasound equipment that will be used to analyze the movement of the diaphragm. These details are important to allow other researchers to reproduce the study.»***

 Thank you for your comment. As you suggested, the commercial references to the ultrasound equipment were added to the main text.

**Comment 5:** ***«I suggest improving the description of the statistical analysis, adding more details about the analyses. In this same item, please add the commercial description of the software to be used»***

*Following your suggestion, *we have improved the subsection on statistical analysis as requested. As you suggested, we have uploaded details about both the analyses and the commercial description of the software to be used.

**Comment 6: *«The study does not mention whether there are conflicts of interest between the authors. It would be interesting to mention if there is any conflict.»***

*Thank you for pointing out. *Here and in the main text, we would like to disclose no conflicts of interest between the authors.

**Comment 7:** ***«I suggest that the authors standardize bibliographic references. There are some non-standard ones.»***

 Thank you for your comment. We apologize for the mistake you pointed out. We have now tried to upload the bibliographic references standardized to the best.
